# Leveraging Advanced Data Analytics to Predict the Risk of All-Cause Seven-Day Emergency Readmissions

**DOI:** 10.7759/cureus.27630

**Published:** 2022-08-03

**Authors:** Mohammed D Aldhoayan, Afnan M Khayat

**Affiliations:** 1 Health Affairs, King Abdulaziz Medical City, Ministry of National Guard – Health Affairs, Riyadh, SAU; 2 Health Informatics, King Saud Bin Abdulaziz University for Health Sciences, Riyadh, SAU

**Keywords:** emergency department, 7-days readmission, machine learning, emergency hospital readmission, prediction model

## Abstract

Introduction

Emergency readmissions have been a long-time, multifaceted, unsolved problem. Developing a predictive model calibrated with hospital-specific Electronic Health Record (EHR) data could give higher prediction accuracy and insights into high-risk patients for readmission. Thus, we need to proactively introduce the necessary interventions. This study aims to investigate the relationship between features that consider significant predictors of at-risk patients for seven-day readmission through logistic regression in addition to developing several machine learning models to test the predictability of those attributes using EHR data in a Saudi Arabia-specific ED context.

Methods

Univariate and multivariate logistic regression has been used to identify the most statistically significant features that contributed to classifying readmitted and not readmitted patients. Seven different machine learning models were trained and tested, and a comparison between the best-performing model was conducted in terms of five performance metrics. To construct the prediction model and internally validate it, the processed dataset was split into two sets: 70% for the training set and 30% for the test set or validation set.

Results

XGBoost achieved the highest accuracy (64%) in predicting early seven-day readmissions. Catboost was the second-best predictive model at 61%. XGBoost achieved the highest specificity at 70%, and all the models had a sensitivity of 57% except for XGBoost and Catboost at 32% and 38%, respectively. All predictive attributes, patient age, length of stay (LOS) in minutes, visit time (AM), marital status (married), number of medications, and number of abnormal lab results were significant predictors of early seven-day readmissions while marital status and number of vital-sign instabilities at discharge were not statistically significant predictors of seven-day readmission.

Conclusion

Although XGBoost and Catboost showed good accuracy, none of the models achieved good discriminative ability in terms of sensitivity and specificity. Thus, none can be clinically used for predicting early seven-day readmission. More predictive variables need to be fed into the model, specifically predictors approximate to the day of discharge, in order to optimize the model’s performance.

## Introduction

Hospital readmission in general and emergency readmission, in particular, has been a long-time unsolved problem. It is considered a critical yet preventable contributor to rising healthcare costs and a surrogate indicator of low-quality care during initial hospitalization [1]. Different methods are constantly evolving to address such problems. Recent advanced techniques of machine learning-based prediction models to predict at-risk patients of readmission have been tapped extensively. Such data-driven predictive models are contributing substantially to revealing insights and detecting complex unknown patterns and hidden relationships in multidimensional healthcare data.

A great deal of previous work attempts to develop predictive models with reported success [2-4]. For instance, Makam et al. have constructed a robust model investigating pneumonia patients’ readmissions [2]. Other studies have reported tailored acute myocardial infarction, heart failure models, and diabetic patients [5-6]. However, although these models achieved good discrimination, they target specific patient populations, limiting their scope and making such comparisons between different models challenging and meaningless to get real informative insights [7]. In contrast to models for specific approaches, various robust models in predicting patients with all-cause readmissions possess higher performance, with an area under the curve (AUC) of 0.877-0.904 and 0.777 in the studies by Jiang et al. [8] and De Giorgi & Fabbian [9], respectively.

Furthermore, although all-cause readmission models have reported higher performance levels, ranging from 0.61 to 0.82 AUC, some of these models still come with certain shortcomings. For example, De Giorgi & Fabbian have developed an all-cause readmission model that achieved a high predictive accuracy with an AUC of 0.777 [9]. However, in their model, they used administrative data that are only available months after patient discharge, which could hinder the model's predictive performance. This issue was pointed out by Makam et al. who indicated that such administrative data would not be available in real-time clinical settings and only available for a subset of the population, which couldn’t be informative enough to take strong interventions prior to discharge across a wide range of at-risk patients [2]. Even more, this kind of data is not useful for models that aim at predicting seven-day readmission, such as this current study, which requires near-discharge data for better prediction. To overcome this obstacle, several studies have developed all-cause readmission models using Electronic Health Record (EHR) data [2-4,10]. For example, Makam et al. have developed an all-cause readmission model for pneumonia patients with EHR data collected from six hospitals and reported an AUC of 0.73 [2].

However, no study has been done to develop a predictive model for seven-day emergency readmission in Saudi Arabia hospitals. Thus, this study aims at analyzing multiple clinical and non-clinical features to understand their relationship and significance to the prediction of at-risk patients for seven-day readmission. Furthermore, this study aims at building a machine learning model to predict all-cause seven-day emergency readmission instead of 30-day readmission.

## Materials and methods

Data collection

A retrospective cohort study was conducted at King Abdul-Aziz Medical City (KAMC) in Riyadh, Saudi Arabia. King Abdulaziz Medical City is one of the Ministry of National Guard Health Affairs' medical cities. This study reviewed all patient encounters (all inpatient visits) to the ED of KAMC aged 13 and above who were discharged from the ED between 1 April 2019 and 31 July 2019. This informed that only patients who had unplanned readmission within seven days of discharge and were readmitted via the emergency department will be included in the study dataset for prediction model development. Patients readmitted through an outpatient setting or clinically admitted for procedures and readmissions that are regarded as planned will be excluded. This study has been exempted by the Internal Review Board (IRB) of King Abdullah International Medical Research Center since the acquired data is de-identified.

Data pre-processing

The total ED admissions before pre-processing were 79875 visits. This study includes patient age, LOS in minutes, time of the original ED visit (AM/PM), number of medications, marital status (married/not-married), number of abnormal lab results, and number of unstable vital signs as predictive attributes. The latest laboratory tests (such as WBC, RBC, hemoglobin (Hgb), hematocrit (HCT), etc.) of each patient were used to calculate the number of abnormal lab results; any missing test data were considered normal lab results. Another attribute generated was vital signs instability at discharge. This was processed using the standard reference range used in medical practice.

Feature selection

Hospital readmission depends on a variety of compound, complex features that differ from one setting to another. Some studies considered sociodemographic status, including, age, marital status, and primary and additional diagnoses [11]. Other studies have included patient clinical characteristics proximal to discharge, such as whether a conditional discharge was signed for the patient, laboratory abnormalities, and vital sign instabilities at discharge [12]. However, the potential predictor variables selected for analysis in this study were based on the grounds of clinical relevance to study objectives. Also, the results from a previous literature review and the availability of clinical data stored in the EHR at KAMC were considered. Univariate and multivariate logistic regression were used as feature selection methods to evaluate the relationship between attributes affecting early readmission.

Machine learning algorithms

There is a variety of classic and modern machine learning models distributed across the literature. In this study, in order to predict readmission within seven days, we experimented with several algorithms using predictive analytics. The first model was developed using random forest (RF) with a maximum depth of two levels and 100 estimators [13]. Second, a logistic regression (LR, aka logit, MaxEnt) classifier was developed that implements regularized logistic regression using the “liblinear” solver [14]. The third was developed using decision trees (DT) with a maximum depth of two levels [15]. The fourth was the naive Bayes (NB) classifier for multivariate Bernoulli models [16]. Neural networks (NN) was the fifth classifier developed, consisting of seven hidden layers with a dropout of 20% to control for overfitting and a learning rate of 0.1. All layers exhibit the Elu activation function except for the last layer, which had the sigmoid activation function for a binary outcome. The sixth model developed was XGBoost (XGB) [17]. Catboost (CB) was developed as the seventh and last model trained on the dataset [18]. It’s important to note that all these models were subjected to multiple hyperparameter tunings to select the best setup for each algorithm built. Each algorithm was trained and validated using the Scikit-learn package in Python version 3.8.12 (https://www.python.org/downloads/release/python-3812/).

Model evaluation

To build the model, the processed dataset was split into two sets: 70 % for the training set and 30% for the test set. Four performance measures, that is, the accuracy, specificity, precision, recall, and AUC metrics, were used to assess the performance of different machine learning models and served as a quantitative means of comparing predictive performance among classifiers.

## Results

Participants

Among the 51099 patients who visited the ER, 14.48% experienced seven-day readmission, and 85.51% didn’t experience seven-day readmission. ED visits between 1 April 2019 and 31 July 2019 data were analyzed. The mean age of patients was 39.84 years. The mean ED length of stay was 198.43 minutes. The demographics are listed in Table 1.

**Table 1:** Characteristics of ED patients

Attribute	Mean (SD)	Range
Patient Age	39.21 (18.60)	13-119
Marital status (Married)	59%	0-1

Characteristics of ED readmissions

Originally, there were 78911 records, out of which 77906 records remain after removing outliers. The number of total patients who revisited ED within seven days was 11286. Patients of age between 20 and 34 years old formed 40.79% of the total number of readmitted patients while patients who were older than 85 years only formed 1.52%. The majority of the patients who were readmitted were originally present at the ED in the PM (57.95%). On average, patients who revisited ED had a LOS of 201.26 minutes while the average ED length of stay was 197.95 for the other class. The characteristics of visits are listed in Table 2.

**Table 2:** Characteristics of visits

Attribute	Mean (SD)	Range
LOS in minutes	198.43 (197.76)	0-1006
Time of ED visit (AM)	43%	0-1
Number of medications	0.35 (4.32)	0-787
Number of abnormal lab results	0.07 (0.44)	0-12
Number of unstable vital signs	0.00 (0.05)	0-3

Analysis

In order to identify the factors that have a significant relationship with seven-day readmission, univariate and multivariate logistic regression models were used with a level of significance at a p-value of 0.05 and an odds ratio. Table 3 represents the factors distributed based on readmission and no readmission.

**Table 3:** Predictive attributes after post-processing data distributed based on readmission and no readmission

Predictive attribute	Readmitted (n= 11286)		Not readmitted (n = 66620)		Univariate analysis		
	Mean	SD	Mean	SD	OR	(95% CI)	P-value
Patient age	39.11	18.57	40.06	19.00	0.96	-0.04 – -0.04	<0.001
Visit time (am)	42%		43%		0.17	-1.83 – -1.77	<0.001
# Medications	0.41	8.03	0.34	3.30	0.78	-0.26 – -0.23	<0.001
# Abnormal lab results	0.06	0.41	0.07	0.44	0.44	-0.89 – -0.77	<0.001
# Unstable vital signs	0.00	0.04	0.00	0.05	0.19	-2.12– -1.12	<0.001
LOS in minutes	201.26	199.22	197.95	197.51	0.99	-0.01– -0.01	<0.001
Married	65%		61%		0.18	-1.74 – -1.69	<0.001

Since the univariate logistic regression showed that all attributes were significantly associated with the outcome, they have been all included in the multivariate analysis. However, the multivariate analysis showed different results. The patient's age, length of stay in ER, time of ED visit, number of medications patient has, and number of abnormal lab results on discharge were significant in contributing to classifying readmitted from those not readmitted. Marital status (Married) and the number of vital signs instability at discharge were not statistically significant predictors of seven-day readmission since the odds show no specific direction. Therefore, it was found that holding other variables constant, the odds of being readmitted is decreased by 3% for each unit increase in the patient age. The odds of being readmitted are decreased by 1% for each minute increase in the length of stay, The odds of being readmitted are 40% lower if the patient is admitted to the ED in the AM. The odds of being readmitted are 1.01 times higher for every extra prescribed medication. The odds of being readmitted are decreased by 6% for each unit increase in the number of abnormal lab results. Detailed results are listed in Table 4.

**Table 4:** Shows the predictive variables that were statistically significant in the model using multivariate logistic regression analysis

Attribute	OR	OR (95% CI)	P>|z|
Patient age	0.97	0.97 – 0.79	<0.001
LOS in minutes	0.99	0.99 – 0.99	<0.001
Visit time (am)	0.60	0.58 – 0.63	<0.001
Marital status (Married)	0.98	0.94 – 1.02	0.38
Number of medications	1.01	1.00 – 1.01	<0.001
Number of abnormal lab results	0.94	0.89 – 0.99	0.02
Number of unstable vital signs	0.87	0.55 – 1.37	0.54

Performance of models

After identifying the factors that are associated with the outcome, several machine learning (ML) models were deployed to investigate the predictability of significant attributes. The detailed results are listed below. However, none of the models, including logistic regression, gave a promising result. The testing accuracy of the models ranges from 0.45 to 0.64. XGBoost (XGB) achieved the highest accuracy (64%) while naive Bayes achieved the lowest accuracy (45%), meaning that the model has the ability to classify only 45% percent of admitted and not readmitted cases correctly. The highest specificity (0.70) was achieved by XGBoost with an AUC of 51%. However, while the XGBoost gave the highest specificity, it doesn’t represent any discriminative power since the ability of the model to identify the positive class (the readmitted in this case) is very low sensitivity (0.32). Catboost achieved the highest AUC of 0.52 with relatively good accuracy (61%) and with moderate discriminative ability, with 0.66 specificity; however, again, its sensitivity is low (0.38). Sensitivity ensures that most patients at high risk of seven-day readmission are correctly identified. Random forest, logistic regression, decision trees, and neural networks achieved almost the same results in all metrics. Table 5 represents the performance comparison of each model developed with related evaluation metrics results.

**Table 5:** Performance comparison of the ML algorithms ML: machine learning

Algorithm	Accuracy	Precision	Specificity	Sensitivity	AUC
DT	0.46	0.15	0.45	0.57	0.51
RF	0.46	0.15	0.45	0.57	0.51
LR	0.46	0.15	0.44	0.57	0.51
NN	0.46	0.15	0.44	0.57	0.51
XGB	0.64	0.16	0.7	0.32	0.51
CB	0.61	0.16	0.66	0.38	0.52
NB	0.45	0.15	0.43	0.57	0.5

## Discussion

Reducing hospital readmission is considered a major challenge to healthcare worldwide. There is a paucity of studies explicitly targeting seven-day readmissions [7,19]. Herein, this paper aims at identifying the significant predictors of at-risk patients for early seven-day ED readmission through logistic regression in addition to developing several ML models to test the predictability of such attributes using EHR data.

To the best of our knowledge, no study has been published targeting the prediction of early seven-day emergency readmission in the Saudi Arabian context. The accuracy performance of our models ranges from 45% to 64%. Among the seven models, we can conclude that XGB is the best of the seven models that were experimented with to predict seven-day readmissions in terms of accuracy (64%) and specificity (0.70). However, XGB did not represent any discriminative power since its sensitivity is the lowest (0.32). Catboost was the second-best model with high accuracy (61%) and specificity (66%), however, again with low sensitivity (0.38). Performance comparison of random forest, logistic regression, decision trees, and neural networks showed moderate discriminative ability. A general pattern can be identified in terms of a model’s performance, which is that no model has given a high discriminative ability in classifying seven-day readmission.

Regarding predictive attributes affecting ED readmission, our findings show that there was a significant reduction in the probability of being readmitted with the increase in patient age. Mostly, patients aged between 20 and 34 constituted the majority of readmitted cases (40.79%). This could be due to the fact that elderly patients are taken more care of in the ED. Regarding ED length of stay, the result showed that the longer patients stay in the ED, the lower the probability of them being readmitted. Our analysis also shows that the patient's time of original visit to the ED significantly affects the outcome. It shows that the patients who visited in the AM have a significantly lower probability of being readmitted than those in the PM. This could be attributed to the smaller number of ED visits during the AM shift because doctors have more capacity to focus on each visit, which gives them a lower probability of being readmitted within seven days of their discharge from the ER.

Patients with an increased number of medications were less susceptible to being readmitted to ED within seven days. This could be evidence that failing to prescribe enough medications to patients who need them could significantly lead to seven-day readmission. Furthermore, if the patient has an increased proportion of abnormal labs, they are less susceptible to being readmitted within seven days. This conclusion is in contrast to what has been reported in studies that characteristics at discharge are more predictive of seven-day readmissions [12]. However, a possible explanation for this might be that the worse lab results during ED visits, care providers are getting those patients more care, reducing their probability of being early readmitted. This can also be seen given that vital signs instability at discharge was not statistically significantly associated with the outcome in our study.

The general findings from this study are that the worse the patient’s condition is when they come to the ED, the more the care provided to individual patients, and the less the probability of readmission, which contradicts what prior research has demonstrated [19-20].

Despite the promise of our study, several important limitations should be taken into consideration. Data preprocessing has consumed a lot of time, which could be avoided with more well-structured and controlled data. More strict policies and procedures must be put in place regarding documentation and structured data entry by care providers to enhance data quality, accuracy, precision, and completeness. There is, therefore, much work to be done in the future since factors associated with hospital readmission are versatile and varied. Many of them are unpredictable. Thus, more predictor variables need to be fed into the model, specifically predictors approximal to the day of discharge, to continuously optimize the performance accuracy of the model. And further advanced data preprocessing and feature selection are needed as well.

Moreover, we want to move to a more complicated model, such as recurrent neural networks, and tune a large number of the model’s hyperparameters. However, these are challenging to interpret but with great potential to boost the predictive accuracy of inferior approaches to predict early readmission. Existing high-performing models were trained in much larger datasets. Thus, future work is required to assess if our models could be improved with larger dataset size. This is a subject for future research as well.

Putting those results in the context of Saudi Arabia, using EHR data, this study has not only developed models for predicting early seven-day readmission but has also generated results that helped us determine and define the subset of features that have a significant impact on patients being readmitted within seven days in Saudi Arabia emergency departments. This characterizes what patient group to target prior to discharge for tailoring better interventions for preventing unplanned readmissions. Moreover, the several issues in the dataset retrieved have shed some light on what can be done to improve them.

## Conclusions

This study has successfully trained and tested seven predictive models to predict the risk of patients’ readmission within the early seven days of their discharge using real-time EHR data. The approach involved feature selection using univariate and multivariate logistic regression analysis. This study showed that contradicting the findings of prior research, the worse the patient’s condition is when they come to the emergency department, the more the care and attention provided to the individual patient, and the less the probability rate of readmission to the ED witnessed, which seems contrary to expectations since this study located no significant association between clinical stability on discharge and early readmissions. Future research is needed to increase the number of predictor variables, specifically predictors approximal to the day of discharge, to continuously optimize the performance of the models.
